# Elucidating the *Pseudomonas aeruginosa* Fatty Acid Degradation Pathway: Identification of Additional Fatty Acyl-CoA Synthetase Homologues

**DOI:** 10.1371/journal.pone.0064554

**Published:** 2013-05-29

**Authors:** Jan Zarzycki-Siek, Michael H. Norris, Yun Kang, Zhenxin Sun, Andrew P. Bluhm, Ian A. McMillan, Tung T. Hoang

**Affiliations:** 1 Department of Microbiology, University of Hawaii at Manoa, Honolulu, Hawaii, United States of America; 2 Department of Molecular Bioscience and Bioengineering, University of Hawaii at Manoa, Honolulu, Hawaii, United States of America; University of Helsinki, Finland

## Abstract

The fatty acid (FA) degradation pathway of *Pseudomonas aeruginosa*, an opportunistic pathogen, was recently shown to be involved in nutrient acquisition during BALB/c mouse lung infection model. The source of FA in the lung is believed to be phosphatidylcholine, the major component of lung surfactant. Previous research indicated that *P. aeruginosa* has more than two fatty acyl-CoA synthetase genes (*fadD*; PA3299 and PA3300), which are responsible for activation of FAs using ATP and coenzyme A. Through a bioinformatics approach, 11 candidate genes were identified by their homology to the *Escherichia coli* FadD in the present study. Four new homologues of *fadD* (PA1617, PA2893, PA3860, and PA3924) were functionally confirmed by their ability to complement the *E. coli fadD* mutant on FA-containing media. Growth phenotypes of 17 combinatorial *fadD* mutants on different FAs, as sole carbon sources, indicated that the four new *fadD* homologues are involved in FA degradation, bringing the total number of *P. aeruginosa fadD* genes to six. Of the four new homologues, *fadD4* (PA1617) contributed the most to the degradation of different chain length FAs. Growth patterns of various *fadD* mutants on plant-based perfumery substances, citronellic and geranic acids, as sole carbon and energy sources indicated that *fadD4* is also involved in the degradation of these plant-derived compounds. A decrease in fitness of the sextuple *fadD* mutant, relative to the Δ*fadD1D2* mutant, was only observed during BALB/c mouse lung infection at 24 h.

## Introduction

*Pseudomonas aeruginosa* is an important human pathogen [1], [2] responsible for myriad of infections of the human body [3]–[11]. This ubiquitous bacterium is also a leading cause of mortality and morbidity in patients with cystic fibrosis (CF) [1], [2].

Phosphatidylcholine (PC), the major component of lung surfactant [12], was suggested as a potential nutrient source for pathogenesis during *P. aeruginosa* infection of the CF lung [13]. The major carbon source within the PC molecule comes from the two highly reduced long-chain fatty acids (LCFA). Many fatty acid degradation (β-oxidation) genes are expressed by *P. aeruginosa* during CF lung infection (e.g. *fadD1*: PA3299, *fadD2*: PA3300, *fadA5*: PA3013, and *fadB5*: PA3014) [13] and mutants defective in the fatty acid (FA) degradation pathway were reported to have decreased fitness during mouse lung infection [14]. A link between FA degradation genes and virulence was also observed [14] and *P. aeruginosa* can chemotax towards FA [15]. Furthermore, FA was shown to modulate type three-secretion system expression in this bacterium [16].

**Figure 1:**
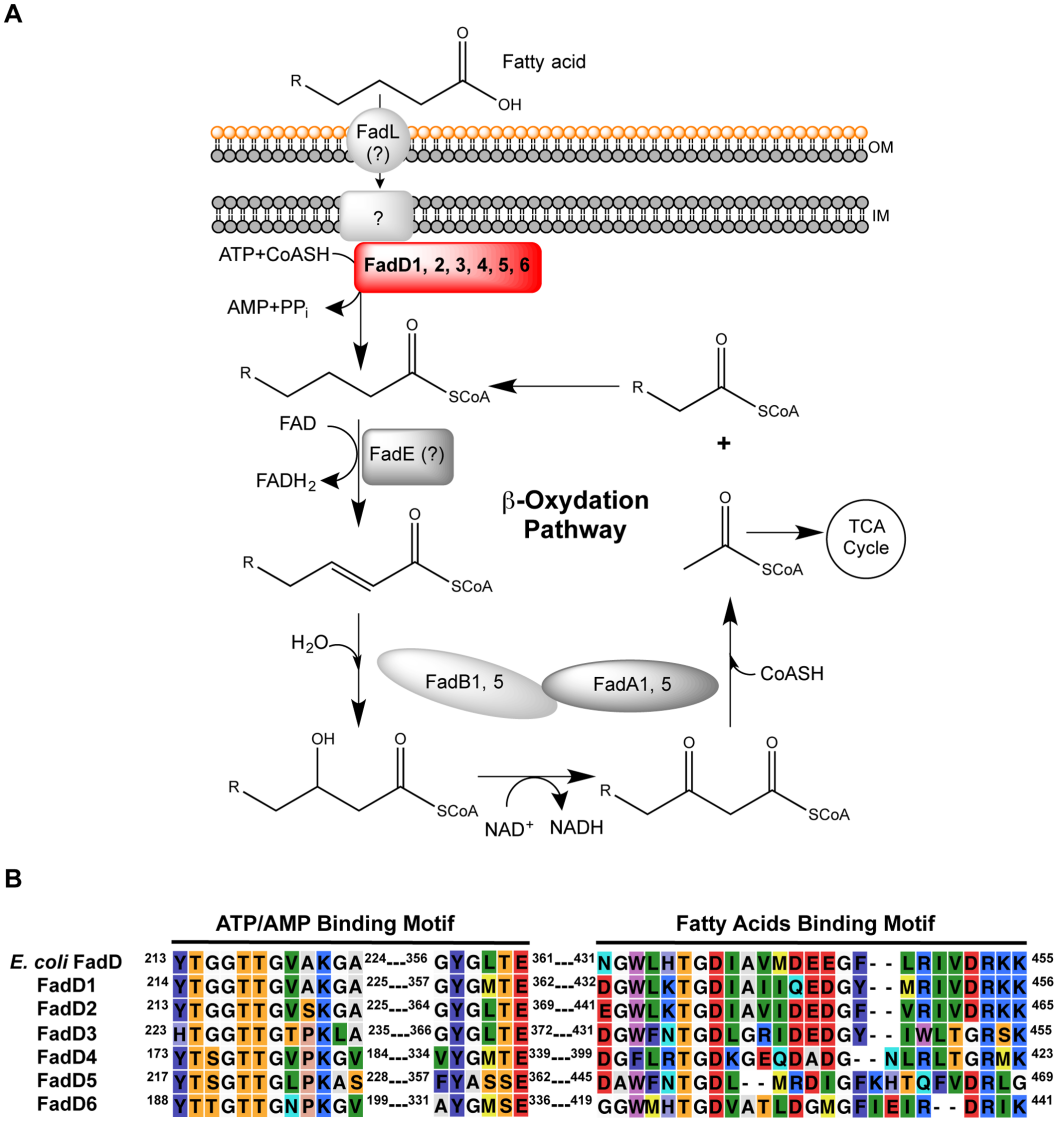
*P. aeruginosa* fatty acid degradation pathway (FA degradation). (A) *P. aeruginosa* FA degradation model was based on the *E. coli* β-oxidation pathway. Known *P. aeruginosa* FA degradation enzyme homologues are indicated by numbers: FadD1 (PA3299), FadD2 (PA3300), FadD3 (PA3860), FadD4 (PA1617), FadD5 (PA2893), FadD6 (PA3924), FadAB1 (PA1736–PA1737), and FadBA5 (PA3013–PA3014). Abbreviations: FadA, 3-ketoacyl-CoA thiolase; FadB, *cis*-Δ^3^-*trans*-Δ^2^-enoyl-CoA isomerase, enoyl-CoA hydratase, 3-hydroxyacyl-CoA epimerase, and 3-hydroxyacyl-CoA dehydrogenase; FadD, fatty acyl-CoA synthetase; FadE, acyl-CoA dehydrogenase; FadL, outer membrane long-chain fatty acid translocase; OM, outer membrane; IN, inner membrane. (B) Alignment of FadD homologues motifs with *E. coli* FadD motifs. Amino acids with similar properties are assigned the same colors using CLC Sequence Viewer 6 software (www.clcbio.com).

Despite the connection between virulence and FA degradation during infections, not all genes involved in *P. aeruginosa* FA degradation are characterized (Fig. 1A). In contrast, genes needed by *Escherichia coli* for aerobic β-oxidation (*fadL*, *fadD*, *fadE*, and *fadBA* [17]–), anaerobic FA degradation (*fadK* and *fadIJ* [21]), and auxiliary genes (*fadH* [22] and *fadM* [23]) are well characterized. For an exogenous FA to be degraded by this pathway, it must first be transported by the membrane transporter (FadL) into the cell [24]. FA is then activated with the use of adenosine triphosphate (ATP) and coenzyme A (CoASH) by FadD (fatty acyl-CoA synthetase, FACS) [19], [25]. The activated FA molecule can then proceed through the β-oxidation pathway (Fig. 1A). In *E. coli*, genes encoding enzymes needed for β-oxidation (*fadL, fadD, fadE, and fadBA*) are repressed in the absence of FAs by the transcriptional regulator FadR. Acyl-CoA of chain length ≥ C_12∶0_ can bind to FadR to induce FA degradation [18], [26], [27] resulting in growth on FA (> C_10∶0_). Cyclic AMP and receptor protein complex levels [28], presence of oxygen [29], and osmotic pressure [30] also affect expression of FA degradation genes in *E. coli*. However, the existence of a central regulator, such as *fadR*, is unknown in *P. aeruginosa*, and only a few *fad*-genes have been found to be regulated by a FA sensor, PsrA [31].

*P. aeruginosa* exhibits greater metabolic capabilities for FA degradation than *E. coli* by growing aerobically on short, medium, and long-chain FAs as sole carbon and energy sources [31]. With a genome of 6.3 Mb, *P. aeruginosa* could potentially have more FA degradation genes than *E. coli* [32], suggesting possible redundancies and a higher level of complexity in this pathway. Three potential *fadL*s have been investigated thus far in *P. aeruginosa* and their exact role in FA transport still remains unclear [15]. Two *fadBA* operon homologues (*fadAB1* and *fadBA5*) have been studied so far. The *fadAB1* (PA1736 and PA1737) operon was shown to be strongly induced by medium-chain fatty acids (MCFA, C_10∶0_ and C_12∶0_) and, to a lesser extent, LCFA (C_14∶0_–C_18∶1_^Δ9^) [33]. The *fadBA5* (PA3014 and PA3013) operon was determined to be involved in LCFA metabolism and to be induced by LCFA, especially oleate (C_18∶1_^Δ9^) [31]. We have recently identified two FACS homologues of *P. aeruginosa*, *fadD1* (PA3299) and *fadD2* (PA3300) [14]. The FadD1 and FadD2 of *P. aeruginosa* were determined to have broad specificity for FA of different chain lengths. FadD1 has preference for LCFA whereas FadD2 has higher activities for shorter chain FAs. *fadD1*, *fadD2,* and *fadD2D1* mutants showed growth defects when grown on minimal media with different length FAs as sole carbon sources. *fadD1* was determined to be induced by LCFA and to be more important for growth on LCFA while *fadD2* was important for growth on short-chain fatty acids (SCFA) and was induced by MCFA. The double mutant *fadD2D1* displayed an impaired ability to grow on PC as a sole carbon source. This growth defect translated into decreased *in vivo* fitness during mouse lung infection, indicating that FadD1 and FadD2 may mediate *P. aeruginosa* replication in the CF lung [14]. However, the double mutant *fadD2D1* was still able to grow on FA, suggesting the involvement of other *fadD* homologues in FA degradation [14].

We surveyed the *P. aeruginosa* genome for additional *fadD* homologues to gain more insight into the degradation of FAs in this bacterium. Four new *fadD* homologues PA1617, PA2893, PA3860, and PA3924 were identified out of 11 potential candidates. Through genetic analyses, their contribution to FA degradation was assessed. The final four candidates were determined to be FACS homologues, but PA1617 (*fadD4*) was found to be the major contributor to FA degradation. Involvement of the newly discovered *fadD4* in catabolism of plant-derived acyclic terpenes suggests that the function of multiple FACS in *P. aeruginosa* is the degradation of compounds closely related to FAs. Growth defect on PC and decreased fitness in mouse lung of the sextuple *fadD* mutant supports the role of FA as a nutrient *in vivo*.

## Results

### Identification of *P. aeruginosa* Fatty acyl-CoA Synthetase Homologues

To identify *fadD* homologues of *P. aeruginosa*, *E. coli* FadD amino acid sequence was compared to *P. aeruginosa* PAO1 ORFs via BLAST [34]. The amino acid sequence of genes obtained in the search were further analyzed for the presence of ATP/AMP [19], [35]–[37] and fatty acid binding motifs [38]. Genes that encode eleven proteins containing amino acid sequences with high degree of similarity to the motifs found in *E. coli* FadD (Fig. S1) were chosen for complementation tests. Identity and similarity of the proteins range from 22% to 31% and from 37% to 52%, respectively (Table S1). When cloned into a high copy number pUC19 vector, only genes encoding PA3860, PA1617, PA2893, and PA3924 were found to complement the *E. coli fadD^−/^fadR^−^* (E2011) strain on minimal medium containing oleate (C_18∶1_^Δ9^) and decanoate (C_10∶0_) (Table S1) and were designated *fadD3*, *fadD4*, *fadD5*, and *fadD6*, respectively. Their ATP/AMP and FA binding motifs show high degree of similarity to those of *E. coli* FadD (Fig. 1B).

All four *P. aeruginosa fadD* genes (*fadD3, fadD4, fadD5,* and *fadD6*) were tested further for their ability to support growth of *E. coli fadD^−/^fadR^−^* (E2011) on various FAs as a single copy on the *E. coli* chromosome. The *E. coli fadD^−/^fadR^−^* double mutant was used to ensure that FadR does not inhibit expression of other *E. coli* β-oxidation enzymes. Mini-Tn7 based complementation vectors were constructed and integrated into the E2011 chromosome at the *att*Tn7 site and resulting strains were tested for growth on FAs (Table 1). As expected, wildtype *E. coli* control strain K-12 showed growth on longer FAs (C_12∶0_–C_18∶1_^Δ9^) but not on the MCFA, C_10∶0_, or SCFAs (C_4∶0_–C_8∶0_). The E2011 and the integrated empty-vector control strain were not able to growth on any of the FAs. E2011 complemented with *E. coli fadD* (*fadD_Ec_*) grew on C_12∶0_–C_18∶1_^Δ9^ comparably to K-12. *P. aeruginosa fadD3*, *fadD4*, *fadD5*, and *fadD6* genes individually allowed E2011 to grow on C_14∶0_–C_18∶1_^Δ9^ to similar levels as K-12. *fadD3* and *fadD6* complemented E2011 to a lesser degree than *fadD4* and *fadD5* on C_12∶0_, and four *fadD* genes supported minimal growth of E2011 on C_10∶0_ to the same level as *fadD_Ec_*. E2011 complemented with *fadD_Ec_*, *fadD3*, *fadD4, fadD5,* or *fadD6* did not grow on C_4∶0_–C_8∶0_, which was in agreement with previous observations that other *E. coli* FA degradation enzymes do not support metabolism of shorter FAs [39].

**Table 1:** Single copy complementation of the *E.coli fadD* mutant with *P. aeruginosa fadD* homologues.

	Growth on different carbon sources								
Strain	C_4∶0_	C_6∶0_	C_8∶0_	C_10∶0_	C_12∶0_	C_14∶0_	C_16∶0_	C_18∶1_^Δ9^	Glu
K12	–	–	–	–	+4	+5	+5	+5	+6
E2011 (*fadD^−/^fadR^−^* )	–	–	–	–	–	–	–	–	+6
E2011/*att*Tn7::miniTn7-Gm^r^	–	–	–	–	–	–	–	–	+6
E2011/*att*Tn7::*fadD_Ec_*	–	–	–	+1	+5	+5	+5	+5	+6
E2011/*att*Tn7::*fadD3*	–	–	–	+1	+3	+5	+5	+5	+6
E2011/*att*Tn7::*fadD4*	–	–	–	+1	+5	+5	+5	+5	+6
E2011/*att*Tn7::*fadD5*	–	–	–	+1	+5	+5	+5	+5	+6
E2011/*att*Tn7::*fadD6*	–	–	–	+1	+2	+5	+5	+5	+6

Strains were grown on 1x M9 medium +1% (w/v) Brij-58 supplemented with 0.2% (w/v) fatty acids or 20 mM glucose (Glu) +0.25 mM IPTG for three days at 37°C.

– indicates no growth on a patch and+denotes growth.

+1 is very little growth whereas +6 is very heavy growth comparable to K12 on glucose at day 3.

### Contribution of *fadD3*, *fadD4*, *fadD5*, and *fadD6* to FA Degradation

To determine the role of the *fadD* homologues (*fadD3*, *fadD4*, *fadD5*, and *fadD6*) in *P. aeruginosa* FA degradation, strains with various combinations of *fadD* mutations were created. To prevent potential masking of phenotypes by *fadD1* and *fadD2*, 15 mutants were constructed in the *P. aeruginosa* PAO1 *ΔfadD1D2* background. Four triple, seven quadruple, four quintuple mutants and one sextuple mutant (Table 2) were tested for growth on C_4∶0_–C_18∶1_^Δ9^ along with wildtype PAO1 and the Δ*fadD1D2* mutant.

**Table 2:** Strains utilized in this study.

Strain	Lab ID	Relevant Properties	Source/reference
***E. coli***			
K-12	E0577	Prototroph	ATCC #23740
*E. coli fadD* ^−/^ *fadR^−^*	E2011	Km^r^; *fadD^−^* (*oldD88*) *fadR*::Km^r^	[14]
E2011/*att*Tn7::miniTn7-Gm^r^	E2665	Gm^r^, Km^r^; E2011 with miniTn7-Gm^r^ vector inserted at *att*Tn7 site	This study
E2011/*att*Tn7::*fadD3*	E2666	Gm^r^, Km^r^; E2011 with *fadD3* inserted at *att*Tn7 site	This study
E2011/*att*Tn7::*fadD4*	E2667	Gm^r^, Km^r^; E2011 with *fadD4* inserted at *att*Tn7 site	This study
E2011/*att*Tn7::*fadD5*	E2799	Gm^r^, Km^r^; E2011 with *fadD5* inserted at *att*Tn7 site	This study
E2011/*att*Tn7::*fadD6*	E2798	Gm^r^, Km^r^; E2011 with *fadD6* inserted at *att*Tn7 site	This study
E2011/*att*Tn7::*fadD_Ec_*	E2385	Gm^r^, Km^r^; E2011 with *fadD_Ec_* inserted at *att*Tn7 site	This study
***P. aeruginosa***			
PAO1	P007	Prototroph	[59]
Δ*fadD4*	P691	PAO1-*fadD4*::*FRT*	This study
Δ*fadD4*/*attB*::*fadD4*	P1041	Tc^r^; PAO1-*fadD4*::*FRT*/*attB*::miniCTX2-*fadD4*	This study
Δ*fadD1D2*	P177	PAO1-Δ*fadD2D1*::*FRT*	[14]
Δ*fadD1D2D3*	P678	PAO1-Δ*fadD2D1*::*FRT*/Δ*fadD3*::*FRT*	This study
Δ*fadD1D2D4*	P696	PAO1-Δ*fadD2D1*::*FRT*/*fadD4*::*mFRT*	This study
Δ*fadD1D2D5*	P246	PAO1-Δ*fadD2D1*::*FRT*/*fadD5*::*FRT*	This study
Δ*fadD1D2D6*	P969	PAO1-Δ*fadD2D1*::*FRT*/*fadD6*::*FRT*	This study
Δ*fadD1D2D3D4*	P698	PAO1-Δ*fadD2D1*::*FRT*/Δ*fadD3*::*FRT*/*fadD4*::*mFRT*	This study
Δ*fadD1D2D3D5*	P768	PAO1-Δ*fadD2D1*::*FRT*/Δ*fadD3*::*FRT*/*fadD5*::*FRT*	This study
Δ*fadD1D2D3D6*	P769	PAO1-Δ*fadD2D1*::*FRT*/Δ*fadD3*::*FRT*/*fadD6*::*FRT*	This study
Δ*fadD1D2D4D5*	P770	PAO1-Δ*fadD2D1*::*FRT*/*fadD4*::*FRT*/*fadD5*::*FRT*	This study
Δ*fadD1D2D4D6*	P771	PAO1-Δ*fadD2D1*::*FRT*/*fadD4*::*mFRT*/*fadD6*::*FRT*	This study
Δ*fadD1D2D5D6*	P722	PAO1-Δ*fadD2D1*::*FRT*/*fadD5*::*FRT*/*fadD6*::*FRT*	This study
Δ*fadD3D4D5D6*	P781	PAO1-Δ*fadD3*::*FRT*/*fadD4*::*mFRT*/*fadD5*::*FRT*/*fadD6*::*FRT*	This study
Δ*fadD1D2D3D4D5*	P772	PAO1-Δ*fadD2D1*::*FRT*/Δ*fadD3*::*FRT*/*fadD4*::*mFRT*/*fadD5*::*FRT*	This study
Δ*fadD1D2D3D4D6*	P773	PAO1-Δ*fadD2D1*::*FRT*/Δ*fadD3*::*FRT*/*fadD4*::*mFRT*/*fadD6*::*FRT*	This study
Δ*fadD1D2D3D5D6*	P726	PAO1-Δ*fadD2D1*::*FRT*/Δ*fadD3*::*FRT*/*fadD5*::*FRT*/*fadD6*::*FRT*	This study
Δ*fadD1D2D4D5D6*	P766	PAO1-Δ*fadD2D1*::*FRT*/Δ*fadD3*::*FRT*/*fadD5*::*FRT*/*fadD6*::*FRT*	This study
Δ*fadD1D2D3D4D5D6*	P767	PAO1-Δ*fadD2D1*::*FRT*/Δ*fadD3*::*FRT*/*fadD4*::*mFRT*/*fadD5*::*FRT*/*fadD6*::*FRT*	This study
Δ*fadD1D2D3D4D5D6*/*mucA^−^*	P973	Cb^r^; P767/*mucA*::pUC18	This study
Δ*fadD1D2D3D4D5D6*/complement	P1021	Gm^r^, Tc^r^; P767/*attB*::miniCTX2-*fadD2D1D4*/*attTn7*::miniTn7-*fadD3-fadD5-fadD6*	This study
Δ*fadD1D2D3D4D5D6*/complement/*mucA^−^*	P1028	Cb^r^, Gm^r^, Tc^r^; P767/*attB*::miniCTX2-*fadD2D1D4*/*att*Tn7:miniTn7-*fadD3-fadD5-fadD6*/*mucA*::pUC18	This study

Abbreviations:

Cb^r^; carbenicilin resistance; *Ec*, *E. coli*; *fadD*, gene encoding fatty acyl-CoA synthetase; Flp, *Sacchaomyces cerevisiae* recombinase; *FRT*, Flp recognition target; Gm^r^, gentamicin resistance; Km^r^, kanamycin resistance; *mucA*, anti-sigma factor, repressor of alginate biosynthesis in *P. aeruginosa*; *Pa*, *P. aeruginosa*; *pheS*, gene encoding a mutated *α-*subunit of phenylalanyl tRNA synthase; Tc^r^, tetracycline resistance.

As expected, all 17 mutant strains grew the same as PAO1 on glucose at 24 h and 96 h (Tables 3 and 4). On C_4∶0_, growth of all mutants was the same as PAO1 indicating that none of the *fadD* homologues contribute to the degradation of this FA or the differences were too small to be detected via plate-based growth assays. Throughout the study, the Δ*fadD3D4D5D6* strain had the same growth as PAO1 on C_6∶0_–C_18∶1_^Δ9^ indicating that FadD1 and FadD2 are most likely providing a majority of FACS activity in *P. aeruginosa* (Tables 3 and 4). No difference in growth was observed between Δ*fadD1D2* strain and Δ*fadD1D2D3*, Δ*fadD1D2D5*, Δ*fadD1D2D6*, Δ*fadD1D2D5D6*, Δ*fadD1D2D3D5*, Δ*fadD1D2D5D6,* or Δ*fadD1D2D3D6* on C_6∶0_–C_18∶1_^Δ9^. There was significantly less growth for Δ*fadD1D2D4* on C_6∶0_–C_18∶1_^Δ9^ at 24 h in comparison to Δ*fadD1D2,* suggesting that *fadD4* is important for degradation of all FAs from C_6∶0_ to C_18∶1_^Δ9^.

**Table 3:** Growth of various *P. aeruginisa fadD* mutants on FAs after 24 h.

	Growth on different carbon sources								
Strain	C_4∶0_	C_6∶0_	C_8∶0_	C_10∶0_	C_12∶0_	C_14∶0_	C_16∶0_	C_18∶1_^Δ9^	Glu
PAO1	+2	+3	+4	+4	+3	+3	+3	+3	+4
Δ*fadD1D2*	+2	+2	+3	+3	+2	+3	+2	+3	+4
Δ*fadD1D2D3*	+2	+2	+3	+3	+2	+3	+2	+3	+4
Δ*fadD1D2D4*	+2	–	–	+1	+1	+1	+1	+1	+4
Δ*fadD1D2D5*	+2	+2	+3	+3	+2	+3	+2	+3	+4
Δ*fadD1D2D6*	+2	+2	+3	+3	+2	+3	+2	+3	+4
Δ*fadD1D2D3D4*	+2	–	–	–	–	+1	+1	+1	+4
Δ*fadD1D2D3D5*	+2	+2	+3	+3	+2	+3	+2	+3	+4
Δ*fadD1D2D3D6*	+2	+2	+3	+3	+2	+3	+2	+3	+4
Δ*fadD1D2D4D5*	+2	–	–	–	–	–	–	+1	+4
Δ*fadD1D2D4D6*	+2	–	–	–	–	+1	+1	+1	+4
Δ*fadD1D2D5D6*	+2	+2	+3	+3	+2	+3	+2	+3	+4
Δ*fadD3D4D5D6*	+2	+3	+4	+4	+3	+3	+3	+3	+4
Δ*fadD1D2D3D4D5*	+2	–	–	–	–	–	–	–	+4
Δ*fadD1D2D3D4D6*	+2	–	–	–	–	+1	+1	+1	+4
Δ*fadD1D2D3D5D6*	+2	+2	+1	+3	+1	+3	+1	+3	+4
Δ*fadD1D2D4D5D6*	+2	–	–	–	–	–	–	–	+4
Δ*fadD1D2D3D4D5D6*	+2	–	–	–	–	–	–	–	+4

Strains were grown on 1x M9 medium +1% (w/v) Brij-58 supplemented with 0.2% (w/v) fatty acids or 20 mM glucose (Glu).

– indicates no growth on a patch and+denotes growth:

+1 is very little growth.

+4 is a heavy growth comparable to PAO1 on glucose at 24 h.

+6 is a very heavy growth comparable to PAO1 on glucose at 96 h.

**Table 4:** Growth of various *P. aeruginosa fadD* mutants on FAs after 96 h.

	Growth on different carbon sources								
Strain	C_4∶0_	C_6∶0_	C_8∶0_	C_10∶0_	C_12∶0_	C_14∶0_	C_16∶0_	C_18∶1_^Δ9^	Glu
PAO1	+4	+6	+6	+6	+6	+6	+6	+6	+6
Δ*fadD1D2*	+4	+4	+4	+4	+4	+4	+4	+4	+6
Δ*fadD1D2D3*	+4	+4	+4	+4	+4	+4	+4	+4	+6
Δ*fadD1D2D4*	+4	+2	+1	+4	+4	+4	+4	+4	+6
Δ*fadD1D2D5*	+4	+4	+4	+4	+4	+4	+4	+4	+6
Δ*fadD1D2D6*	+4	+4	+4	+4	+4	+4	+4	+4	+6
Δ*fadD1D2D3D4*	+4	–	–	+2	+2	+4	+4	+4	+6
Δ*fadD1D2D3D5*	+4	+4	+4	+4	+4	+4	+4	+4	+6
Δ*fadD1D2D3D6*	+4	+4	+4	+4	+4	+4	+4	+4	+6
Δ*fadD1D2D4D5*	+4	–	–	+1	+3	+3	+4	+4	+6
Δ*fadD1D2D4D6*	+4	–	–	+4	+4	+4	+4	+4	+6
Δ*fadD1D2D5D6*	+4	+4	+4	+4	+4	+4	+4	+4	+6
Δ*fadD3D4D5D6*	+4	+6	+6	+6	+6	+6	+6	+6	+6
Δ*fadD1D2D3D4D5*	+4	–	–	–	–	–	+3	+3	+6
Δ*fadD1D2D3D4D6*	+4	–	–	+2	+2	+4	+4	+4	+6
Δ*fadD1D2D3D5D6*	+4	+4	+4	+4	+4	+4	+4	+4	+6
Δ*fadD1D2D4D5D6*	+4	–	–	+1	+3	+1	–	+1	+6
Δ*fadD1D2D3D4D5D6*	+4	–	–	–	–	–	–	–	+6

Strains were grown on 1x M9 medium +1% (w/v) Brij-58 supplemented with 0.2% (w/v) fatty acids or 20 mM glucose (Glu).

– indicates no growth on a patch and+denotes growth:

+1 is very little growth.

+4 is a heavy growth comparable to PAO1 on glucose at 24 h.

+6 is a very heavy growth comparable to PAO1 on glucose at 96 h.

Addition of *fadD3*, *fadD5*, or *fadD6* mutation to Δ*fadD1D2D4* strain in a quadruple mutant combination resulted in larger deficiencies in growth on FAs in comparison to the triple Δ*fadD1D2D4* mutant (Tables 3 and 4), indicating that *fadD3*, *fadD5*, and *fadD6* also take part in FA degradation and suggesting the dominance of FadD4 over these homologues. The Δ*fadD1D2D3D4*, Δ*fadD1D2D4D5*, and Δ*fadD1D2D4D6* strains showed no growth on C_6∶0_ and C_8∶0_, even after four days, in contrast to the Δ*fadD1D2D4* mutant (Table 4), indicating that *fadD3*, *fadD5*, and *fadD6* are involved in the degradation of these FAs.

All quintuple mutants exhibited some level of growth on several FAs after 96 h (Table 4), whereas no growth was present for the sextuplet mutant combination (Δ*fadD1D2D3D4D5D6*), indicating that all four new *fadD* homologues contribute to FA degradation and that only six aerobic FACS genes are likely present in *P. aeruginosa*. Quintuple mutants with both *fadD4* and *fadD5* mutations (Δ*fadD1D2D3D4D5* and Δ*fadD1D2D4D5D6*) were most deficient in FA degradation (Table 3). Growth patterns of the four quintuple mutants after 96 h (Table 4) suggest that *fadD4*, besides *fadD1* and *fadD2*, is much more important for FA degradation than *fadD3*, *fadD5*, and *fadD6* combined, and *fadD5* contributes to FA degradation more than *fadD3* and *fadD6*. Furthermore, by comparing the phenotypes of double, triple, and quadruple mutants at two time points (Tables 3 and 4) a hierarchy of contributions of *fadD* homologues to the degradation of different chain-length FAs can be assigned as follows: i) FadD4 degrades C_6∶0_–C_18∶1_^Δ9^ (Δ*fadD1D2D4* versus Δ*fadD1D2* in Table 3); ii) FadD5 degrades C_6∶0_–C_14∶0_ (Δ*fadD1D2D4D5* versus Δ*fadD1D2D4* in Tables 3 and 4); iii) FadD3 degrades C_6∶0_-C_12∶0_ (Δ*fadD1D2D3D4* versus Δ*fadD1D2D4* in Tables 3 and 4); and iv) FadD6 degrades C_6∶0_–C_12∶0_ (Δ*fadD1D2D4D6* versus Δ*fadD1D2D4* in Tables 3 and 4).

### *fadD1* and *fadD2* in Comparison to *fadD3*, *fadD4*, *fadD5*, and *fadD6*

**Figure 2:**
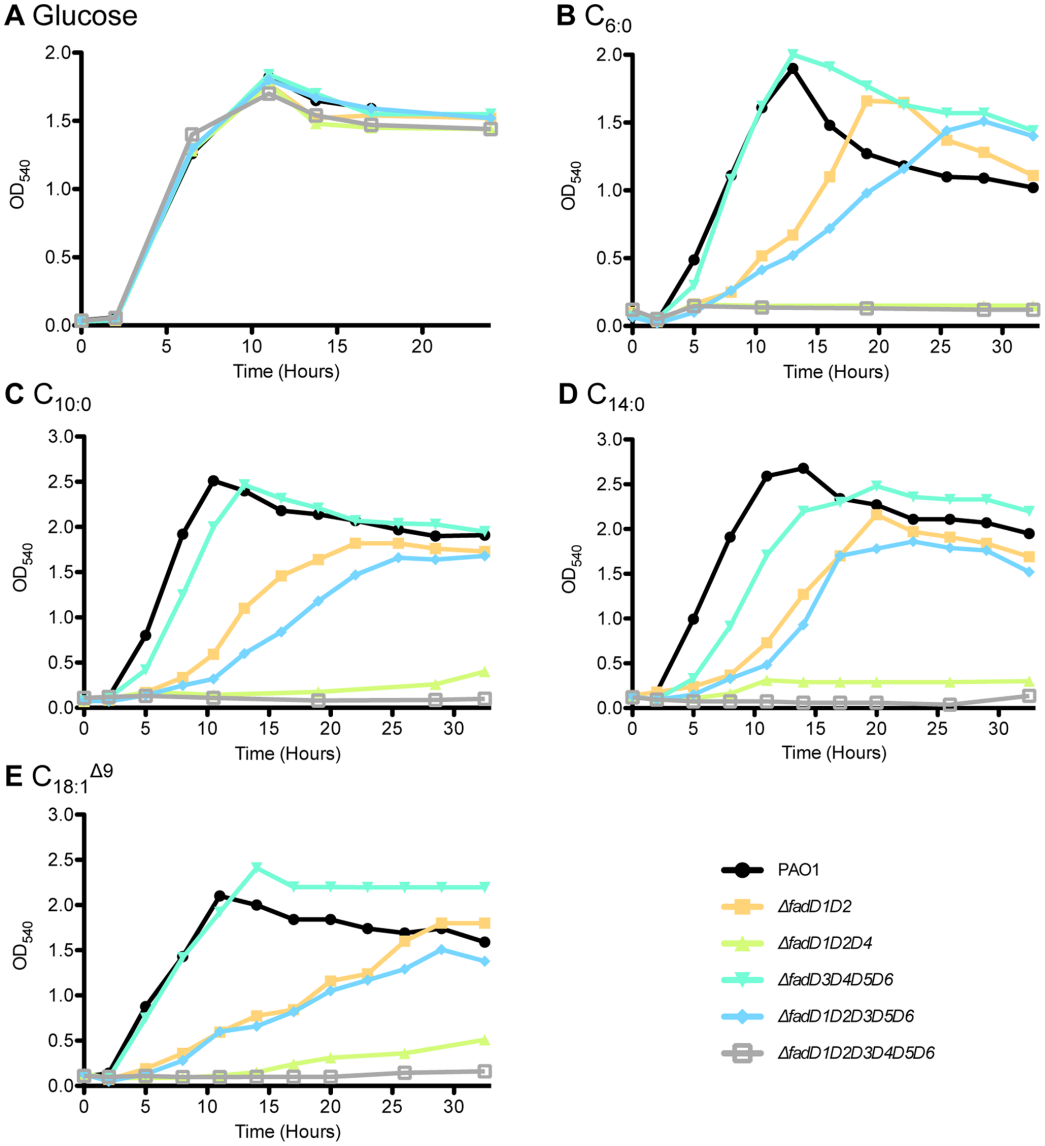
*fadD* mutants and growth on FAs. Various strains were grown on glucose (A), C_6∶0_ (B), C_10∶0_ (C), C_14∶0_ (D), and C_18∶1_^Δ9^ (E) to investigate further the role of *fadD4* in FA degradation in comparison to rest of homologues. These growth curves demonstrate the hierarchical dominance of *fadD1*, *fadD2* and *fadD4* over other *fadD*s. Growth experiments were performed twice and representative curves are shown.

The growth phenotypes of various combinatory mutants on FAs indicated that out of the newly discovered FACS genes (*fadD3*, *fadD4*, *fadD5*, and *fadD6*) *fadD4* is most important for FA degradation (Tables 3 and 4), in addition to *fadD1* and *fadD2* [14]. To investigate further the contribution of *fadD4* to FA degradation in comparison to *fadD1* and *fadD2*, growth curve experiments were performed on SCFA, MCFA, and LCFAs with Δ*fadD1D2D4*, Δ*fadD3D4D5D6*, Δ*fadD1D2D3D5D6*, and Δ*fadD1D2D4D3D5D6* mutants along with PAO1 and Δ*fadD1D2* strains (Fig. 2). The growth experiments on FAs were conducted up to 30 h, which was sufficient to distinguish differences in growth patterns between various strains. The growth rates calculated from growth curves in Fig. 2 are presented in Table S3. The Δ*fadD1D2* mutant strain had impaired growth in comparison to PAO1 on FAs (Fig. 2B–2E). The phenotype of Δ*fadD1D2D3D5D6* in C_6∶0_–C_18∶1_^Δ9^ (Fig. 2B–2E) was characterized by lower final optical density (OD) and/or longer lag phase than Δ*fadD1D2*, indicating that *fadD3*, *fadD5*, and *fadD6* also contribute to FA degradation. In comparison to Δ*fadD1D2* and Δ*fadD1D2D3D5D6*, Δ*fadD1D2D4* exhibited very small amounts of growth, and no increase in turbidity was observed for Δ*fadD1D2D3D4D5D6* on FAs (Fig. 2B–2E). The Δ*fadD3D4D5D6* mutant had almost identical growth in comparison to PAO1 in C_6∶0_ and C_18∶1_^Δ9^ (Fig. 2B and 2E). In C_10∶0_ and C_14∶0_ Δ*fadD3D4D5D6* showed a similar final OD as PAO1 but longer lag phase (Fig. 2C and 2D). These data indicate that, although the activity of FadD4 is masked by the dominance of FadD1 and FadD2, the FadD4 plays a significant role in the degradation of FAs in *P. aeruginosa*.

### Role of *fadD* Homologues in the Utilization of Plant-derived Acyclic Terpenes

**Figure 3:**
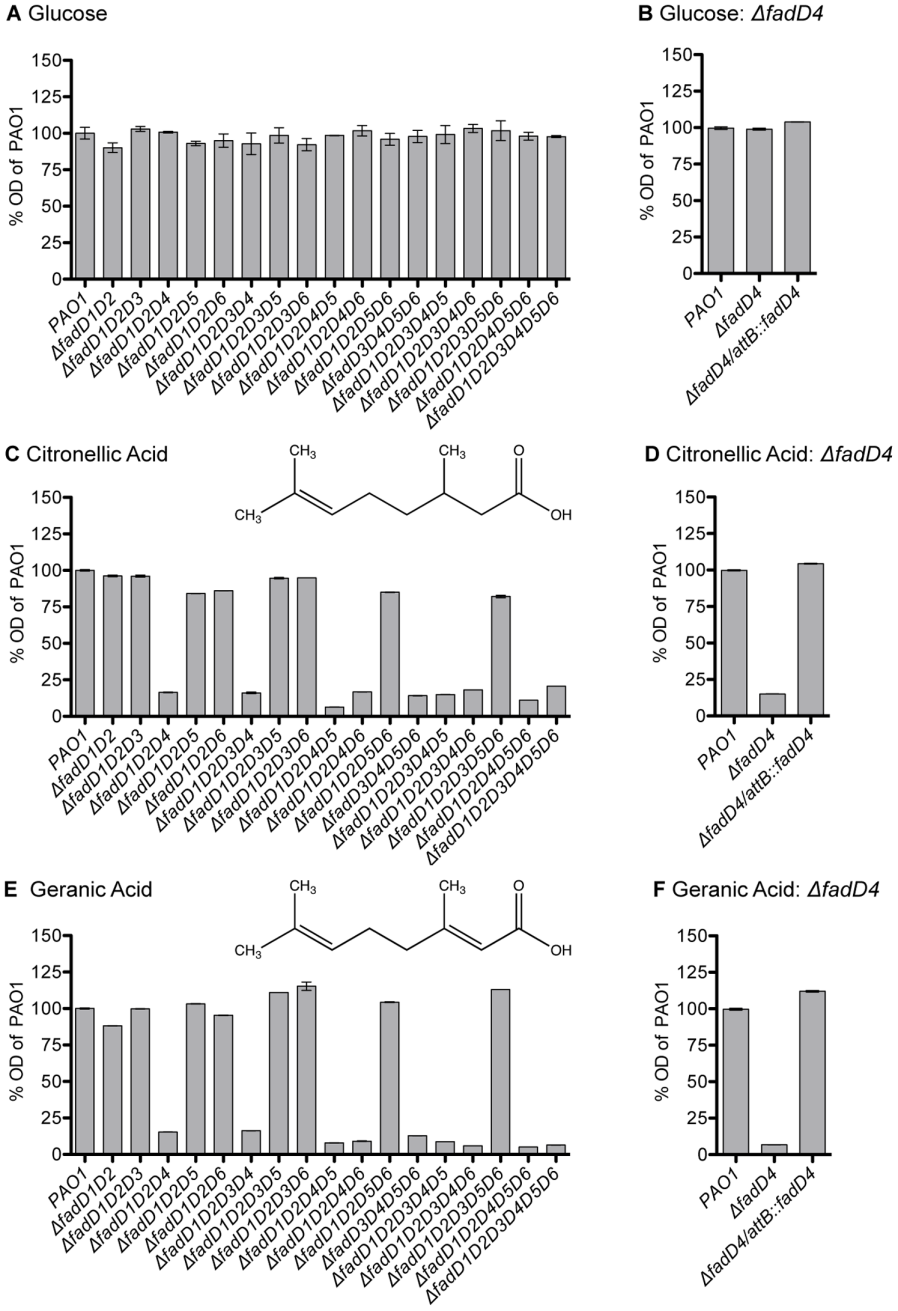
Growth phenotypes of various *fadD* homologues mutants on acyclic terpenes. Strains were grown in liquid 1x M9 medium +1% (w/v) Brij-58 supplemented with 20 mM glucose, 0.1% (w/v) of citronellic acid, or 0.1% (w/v) geranic acid at 30°C. Optical densities (ODs) of cultures were measured and compared to PAO1 at day one (A, C, and E). Growth of Δ*fadD4* mutant and Δ*fadD4*/*attB*::*fadD4* complement strain in different carbon source were compared to PAO1 and ODs from day six are presented (B, D, and F). Results shown are from representative experiments that were performed twice by measuring triplicate cultures.

One of the *P. aeruginosa fadD* homologues, *fadD5* (PA2893; *atuH*), was proposed to be part of the acyclic terpenes utilization (ATU) pathway and to contribute to degradation of citronellol and geraniol (perfumery compounds found in plants) by activating citonellic acid (CA) and geranic acid (GA) through addition of CoASH [40]. However, mutation of PA2893 alone did not abolish growth on acyclic terpenes possibly suggesting the involvement of other homologue(s) [40]. To determine the role of *fadD5* and other *fadD* homologues in degradation of acyclic terpenes as plant-derived nutrient sources, we grew PAO1 along with 17 combinatory *fadD* mutants in 1x M9 minimal media +1% (w/v) Brij-58 with 0.1% (w/v) of CA or GA (Fig. 3). All strains had similar OD measurements after one day of growth on glucose (Fig. 3A). After 24 h, all nine strains with the *fadD4* mutation (triple, quadruple, quintuple, and sextuple combinations) had significantly lower OD for both compounds in comparison to PAO1 (20% or less) (Fig. 3C and 3E). All other mutants had comparable growth to PAO1 in CA and GA (82%–96% and 88%–115%, respectively). None of the strains with *fadD4* mutations had higher OD in CA or GA at day six, than at day one, and the remainder of the mutants grew the same as PAO1 (Fig. S2). Since only strains with *fadD4* mutations exhibited growth defects in CA and GA, involvement of FadD4 in degradation of these compounds was further investigated using the single *fadD4* mutant (Fig. 3D and 3F). Single copy complementation returned growth of the Δ*fadD4* mutant to PAO1 levels indicating that *fadD4* is responsible for the majority of CA and GA degradation.

### *fadD3*, *fadD4*, *fadD5*, and *fadD6* and Virulence in *P. aeruginosa*

A link between *fadDs* and production of virulence factors was previously observed in *P. aeruginosa* [14]. To determine if newly discovered homologues modulate virulence, single unmarked mutants Δ*fadD3*, Δ*fadD4*, Δ*fadD5*, Δ*fadD6*, along with Δ*fadD1D2D3D4D5D6* strain and its complement were tested for production of proteases, lipases, phospholipases, and rhamnolipids. No difference in production of these virulence determinates was observed between PAO1 and all strains tested (data not shown).

### Involvement of New *fadD* Homologues in PC Degradation and *in vivo* Growth

**Figure 4:**
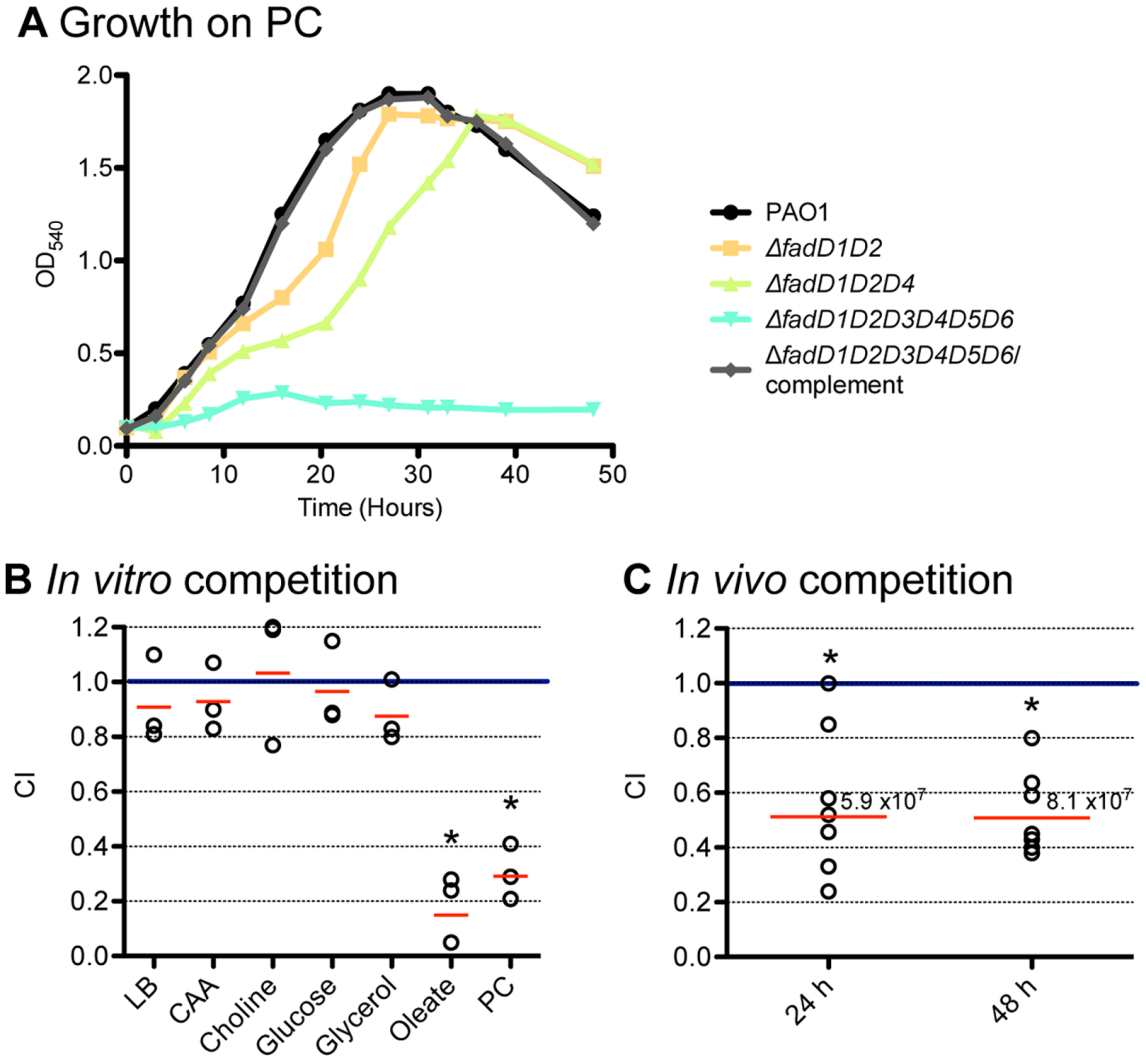
Growth characteristics on PC and competition studies of *fadD* sextuple mutant. (A) PAO1 and several mutant strains were individually grown on PC. Growth curves were performed twice and representative results are shown. (B) *In vitro* competition between Δ*fadD1D2D3D4D5D6* and its competitor, Δ*fadD1D2D3D4D5D6*/complement (P1021), in different growth media after 24 h. (C) *In vivo* competition between Δ*fadD1D2D3D4D5D6*/*mucA^−^* (P973) and its competitor, Δ*fadD1D2D3D4D5D6*/complement/*mucA^−^* (P1028), in BALB/c mice lungs. Seven mice for each time point were inoculated with 6 x10^6^ CFU/mouse. The geometric mean of competitive indices (CI) from each group is marked by red line. Mutant strain is less competitive than complement when CI<1. Total average lung CFU recovered form mice in each group are indicated above red line. * P<0.05 based on one sample *t* test.

Our previous study indicated that the Δ*fadD1D2* mutant had a decreased ability to degrade PC and was less fit in BALB/c mice lungs [14]. We hypothesized that the sextuple *fadD* mutant, which does not grow on FAs, would exhibit impaired growth on PC and have significantly decreased *in vivo* fitness. We first investigated the role of the four newly discovered FACS in PC degradation (Fig. 4A). Before death phase, Δ*fadD1D2* exhibited slower growth rate and lower final turbidity than PAO1. Δ*fadD1D2D4* had a longer lag phase in comparison to Δ*fadD1D2* before reaching a similar OD, implying that *fadD4* contributes to degradation of PC. The Δ*fadD1D2D3D4D5D6* mutant further exhibited a significant growth defect on PC. The large differences in growth rate and final OD between the sextuple mutant and Δ*fadD1D2D4* suggest that not only *fadD4* but also *fadD3*, *fadD5*, and *fadD6* are required for growth on PC, which contains a mixture of FA chain lengths.

When *in vitro* competition studies were conducted on the sextuple *fadD* mutant and its competitor the complemented sextuple *fadD* mutant, mutation of all six FACS genes did not affect fitness when the bacteria were grown in rich Luria Bertani (LB) medium, and minimal medium supplemented with casamino acids, glucose, glycerol, and choline (Fig. 4B). In contrast, the *in vitro* competitive index (CI) in oleate (C_18∶1_^Δ9^) and PC were low (∼0.15 and ∼0.3, respectively) indicating that Δ*fadD1D2D3D4D5D6* has a growth disadvantage on these carbon sources. The *in vivo* competition study showed that the sextuple *fadD* mutant was out numbered by its complement (Fig. 4C). An almost 10-fold increase in CFU per lung above inoculum (6 x10^6^) was observed for both time points indicating bacterial replication *in vivo*. At 24 h, the amount of the sextuple *fadD* mutant was half of its complement, which is lower than the reported CI for the Δ*fadD1D2* mutant at 24 h [14]. Even at 48 h the CI was significantly lower than 1, indicating that deletion of *fadD* genes decreases *in vivo* fitness of sextuple *fadD* mutant.

## Discussion

Previous research on *fadD1* and *fadD2* indicated that more than two FACS genes are present in *P. aeruginosa* [14]. In this study, we focused on identification of additional *fadD* homologues. Four genes, *fadD3*, *fadD4*, *fadD5*, and *fadD6* (PA3860, PA1617, PA2893, and PA3924, respectively) were found to encode FACS (Tables S1 and 1). Each of these genes contributes at a varying degree to FA degradation (Tables 3 and 4). Surprisingly, none of the new *fadD*s were involved in degradation of butyrate (C_4∶0_; Table 3). It is possible that other unidentified genes with acyl-CoA synthetase functions are responsible for growth on C_4∶0_. Butyrate could also be processed through the acetoacetate degradation pathway (*ato*), an alternative pathway for degradation of SCFA [41]. This could be possible since two homologues of both of *E. coli* acetoacetyl-CoA transferase complex proteins, AtoA and AtoD, are present in *P. aeruginosa*: PA2000 (identity 45% and similarity 62%), PA0227 (identity 28% and similarity 62%), PA1999 (identity 40% and similarity 64%), and PA5445 (identity 33% and similarity 55%), respectively.

Growth studies with various mutants using FAs as sole carbon and energy sources indicated that FACS homologues are not of equal physiological significance and that there are disparities in importance and FA preference between them. *fadD1* and *fadD2*, along with *fadD4*, are responsible for almost all FA degradation and dominate over other homologues. When *fadD1* and *fadD2* are inactivated, the majority of growth on SCFAs, MCFAs and LCFAs is due to *fadD4* (Tables 3 and 4, Fig. 2). In comparison, *fadD3*, *fadD5*, and *fadD6* have small contributions to overall growth on FAs and their individual involvement can be only observed when *fadD1*, *fadD2* and *fadD4* are absent (Table 4). This is not unprecedented, since *Pseudomonas putida* FadD2 is only active when FadD1 is not present [42]. It could be possible that gene(s) ruled out by screening in *E. coli* for growth on LCFA (Table S1), might be involved in SCFA and/or MCFA degradation. However, lack of growth for the sextuple *fadD* mutant on C_6∶0_–C_18∶1_^Δ9^ (Table 4) strongly indicates that *P. aeruginosa* has a total of six aerobic FACS genes.

*P. aeruginosa* is commonly found in soil, water, and on plant surfaces [43]–[45] and it is known to degrade over 70 different organic substances such as aromatic compounds, organic acids (e.g. isovalerate), alcohols, and acyclic terpenes (e.g., citronellol and geraniol) [44]. Sources of nutrients for pseudomonads on plant surfaces have not been determined. Citronellol and geraniol (perfumery compounds and possible bacterial nutrient sources found in plants) are degraded through the acyclic terpene utilization (ATU) pathway, β-oxidation pathway, and leucine/isovalerate utilization pathway [40], [46]. The *fadD5* (PA2893 or *atuH*) was proposed to be part of ATU and to be involved in activation of the CA and GA intermediates of the pathway. However, *fadD5* was confirmed experimentally not to be part of ATU, and other homologues were thought to be also involved and to ‘mask’ the phenotype [40]. We investigated the possible role of *fadD* homologues in the degradation of acyclic terpenes, and we reasoned that combination of various *fadD* mutations would allow involvement of FACS homologues in ATU to be assessed. Surprisingly, *fadD5* along with *fadD1*, *fadD2*, *fadD3*, and *fadD6* had minimal if any contributions to the degradation of CA and GA (Fig. 3). Interestingly, *fadD5* is located right next to genes known to be involved in ATU and seems to be the last gene in *atuABCDEFGH* cluster [40]. On the other hand, *fadD4* is not only involved in ATU but it is almost solely responsible for degradation of these compounds as can be observed from growth phenotypes of the single *fadD4* mutant and its complement (Fig. 3D and 3F). Notably, homologues of *fadD4* with high similarity are present in *Pseudomonas fluorescens* (e.g., Pfl01_4205 in Pf0-1, 72% identity and 84% similarity), *Pseudomonas protegens* (e.g., PFL_1744 in strain Pf-5, 71% identity and 82% similarity), and *Pseudomonas mendocina* (e.g., MDS_2302 in strain NK-01, 75% identity and 87% similarity) and some strains of these pseudomonads are known to degrade acyclic terpenes [40], [47].

The ability of *P. aeruginosa* to degrade lipids and FAs, especially the main component of lung surfactant PC, has been linked to replication of this opportunistic pathogen during infection of CF patients’ lungs [13]. Previously, we determined that Δ*fadD1*, Δ*fadD2,* and double Δ*fadD1D2* mutants have decreased fitness in BALB/c mice due to their deficiencies in degradation of FAs and PC [14]. We hypothesized that *P. aeruginosa* strains with greater defects in utilization of FAs and PC *in vitro* will have larger disadvantages during *in vivo* growth. Δ*fadD1D2D3D4D5D6* mutant exhibited the most significant growth defect in FAs and PC (Fig. 2, 4A and 4B), and similar level of virulence factors (i.e. proteases, hemolysins, lipases) production was observed between sextuple *fadD* mutant, its complement, and PAO1 (data not shown). The Δ*fadD1D2D3D4D5D6* mutant had some decrease of *in vivo* fitness in comparison to the Δ*fadD1D2* at 24 h (Fig. 4C and [14]); but at 48 h, Δ*fadD1D2D3D4D5D6* mutant was not less fit in mice lungs than Δ*fadD1D2* mutant. This latter result was surprising, as the impaired ability to utilize PC did not result in a more dramatic phenotype *in vivo* at 48 h (Fig. 4C). There are several possibilities, which could account for this unexpected phenotype. The sextuple mutant could utilize *in vivo* other constituents of PC such as choline and glycerol later in the infection. Additionally, pulmonary surfactants are composed of 10% proteins [48] and amino acids were suggested to be used by *P. aeruginosa* during lung infection [49] and could serve as an alternative nutrient source for sextuple *fadD* mutant. Other FACS genes (i.e. anaerobic which we could not identify because of limitations of our aerobic *in vitro* screening method) could be important for *in vivo* growth.

In summary, we have identified four additional FACS homologues of *P. aeruginosa* and determined their involvement in degradation of different FAs. The dual catabolic function of *fadD4* (PA1617) for FAs and acyclic terpenes exemplifies the interconnection of metabolic pathways and multiple roles that FACS homologues play in this ubiquitous bacterium. Our *in vivo* data show that nutrient acquisition during lung infection is a complicated process, involving alterative pathways that require further investigation. Knowledge of all *fadD* genes needed for FA degradation significantly increases our understanding of the FA degradation pathway and its importance for *in vivo* replication of *P. aeruginosa*.

## Materials and Methods

### Ethics Statement

All animal experiments were approved by University of Hawaii at Manoa Institutional Animal Care and Use Committee (protocol no. 06-023-6) and were conducted in compliance with the NIH (National Institutes of Health) Guide for the Care and Use of Laboratory Animals.

### Bacterial Strains and Growth Media

Strains and plasmids utilized in this study are listed in Tables 2, S2, and 5, respectively. All *P. aeruginosa* mutants constructed and utilized in this study are derived from strain PAO1. *E. coli* E1869 strain (Table S2) was routinely used for cloning and *E. coli* Δ*asd* or Δ*dapA* strains (E464, E1353, and E2072, Table S2) were used for mobilization of plasmids as described previously [50]. *E. coli* and *P. aeruginosa* strains were cultured in rich and minimal media as described by Kang *et al.* [14] unless indicated otherwise. Fatty acids stocks were prepared as previously described [31].

**Table 5:** Plasmids used in this study.

Plasmid	Lab ID	Relevant Properties	Source/reference
miniCTX2	E0076	Tc^r^; *P. aeruginosa* site specific integration vector	[58]
miniCTX2-*fadD2D1*	E2143	Tc^r^; *fadD2D1* cloned into miniCTX2	[14]
miniCTX2- *fadD2D1D4*	E2811	Tc^r^; *fadD4* gene cloned into miniCTX2-*fadD2D1*	This study
miniCTX2-*fadD4*	E2589	Tc^r^; *fadD4* gene cloned into miniCTX2	This study
miniTn7-Gm^r^	E2643	Ap^r^, Gm^r^; pUC18R6Kmini-Tn7 [52] with *FRT8*-Gm^r^ cassette and *lac* promoter cloned	Laboratory collection
miniTn7-*fadD3*	E2645	Ap^r^, Gm^r^; *fadD3* cloned into miniTn7-Gm^r^	This study
miniTn7-*fadD4*	E2647	Ap^r^, Gm^r^; *fadD4* cloned into miniTn7-Gm^r^	This study
miniTn7-*fadD5*	E2793	Ap^r^, Gm^r^; *fadD5* cloned into miniTn7-Gm^r^	This study
miniTn7-*fadD6*	E2794	Ap^r^, Gm^r^; *fadD6* cloned into miniTn7-Gm^r^	This study
miniTn7-*fadD_Ec_*	E2378	Ap^r^, Gm^r^; *E. coli fadD* cloned into miniTn7-Gm^r^	This study
miniTn7-PA3860	E2377	Ap^r^, Gm^r^; *fadD3* with native *rbs* cloned into miniTn7-Gm^r^	This study
miniTn7-PA3924	E2854	Ap^r^, Gm^r^; *fadD6* with native *rbs* cloned into miniTn7-Gm^r^	This study
miniTn7-*fadD3-fadD5-fadD6*	E2860	Ap^r^, Gm^r^; *fadD3, fadD5,* and *fadD6* with native *rbs* cloned into miniTn7-Gm^r^	This study
pCD13SK-*flp-oriT*	E0783	Sp^r^; suicidal Flp-expressing plasmid	[33]
pET15b	E0047	Ap^r^; T7 expression vector	Novagen
pET15b-*fadD3*	E2658	Ap^r^; pET15b with *fadD3* gene	This study
pET15b-*fadD5*	E1127	Ap^r^; pET15b with *fadD5* gene	This study
pET15b-*fadD6*	E2790	Ap^r^; pET15b with *fadD6* gene	This study
pET28a	E0158	Km^r^; T7 expression vector	Novagen
pET28a-*fadD4*	E2644	Km^r^; pET28a with *fadD4* gene	This study
pEX18T	E0055	Ap^r^; gene replacement vector	[53]
pEX18T-*fadD3*-Gm^r^ -*pheS_Pa_*	E2438	Ap^r^, Gm^r^; Gm^r^-*pheS_Pa_-FRT* cassette inserted into *fadD3*	This study
pEX18T-*fadD4*-Gm^r^ -*pheS_Pa_*	E2506	Ap^r^, Gm^r^; Gm^r^ *-pheS_Pa_-mFRT* cassette inserted into *fadD4*	This study
pEX18T-*fadD5*-Gm^r^	E0828	Ap^r^, Gm^r^; Gm^r^-*FRT* cassette inserted into *fadD5*	This study
pEX18T-*fadD6*-Gm^r^	E1476	Ap^r^, Gm^r^; Gm^r^-*FRT* cassette inserted into *fadD6*	This study
pFLP2	E0067	Ap^r^; broad-host range Flp expressing plasmid	[53]
pmFRT-Gm^r^-*pheS_Pa_*	E2382	Ap^r^, Gm^r^; plasmid with Gm^r^ *-pheS_Pa_*-*mFRT* cassette	Laboratory collection
pPS856	E0050	Ap^r^, Gm^r^; plasmid with Gm^r^-*FRT* cassette	[53]
pTNS2	E1189	Ap^r^; helper plasmid for Tn7 transposition system	[52]
pUC18-'*mucA*'	E1907	Ap^r^; pUC18 with internal fragment of *mucA* cloned	[14]
pUC19	E0014	Ap^r^; cloning vector	[60]
pUC19-PA1617	E2472	Ap^r^; PAO1 PA1617 gene cloned into pUC19	This study
pUC19-PA3860	E2356	Ap^r^; PAO1 PA3860 gene cloned into pUC19	This study
pwFRT-Gm^r^-*pheS_Pa_*	E2380	Ap^r^, Gm^r^; plasmid with Gm^r^ -*pheS_Pa_-FRT* cassette	Laboratory collection

Abbreviations:

Ap^r^, ampicillin resistance; *lac*, *E. coli* lactose operon; *rbs*, ribosomal binding site; Sp^r^, streptomycin resistance.

### General Molecular Techniques

Molecular techniques were performed as previously described [50]. Oligonucleotides (Table 6) were synthesized through Integrated DNA Technologies.

**Table 6:** Oligonucleotides primers utilized in this study.

Primer number and name	Sequence^a^
438; PA2893-BamHIb, c	5'-CAGTAGGATCCCACGGTGCTCAGAAGCGGT-3'
512; PA3924-BamHIb, c	5'-TGCTTGGGATCCGGGCGTTTCGGCGGTGTA-3'
1093; EcfadD-down-BamHI^b^	5′-AACGGGATCCTCAGGCTTTATTGTC-3′
1109; PA3924-NdeI^b^	5'-GTGTACGCCATATGCTGAATACCC-3'
1151; PA1221 BamHI-up^d^	5'-ACCGTGGATCCATTCTCATCGCTTTTCTCTC-3'
1152; PA1221 BamHI-down^d^	5'-AGCGCGTTTTCGTCGGCGAAGGATCCGACT-3'
1153; PA2557 BamHI-up^d^	5'-TGGGCGGATCCGCCTCTTGCGTTTACCTT-3'
1154; PA2557 BamHI-down^d^	5'-GAAAGCGAAGCTGCCACTCTTCAGGATCCGCGA GT-3'
1155; PA3860 BamHI-up^d^	5'-GAACGGGATCCAGTGTAAAGCATGTTGCCAG-3'
1156; PA3860 BamHI-downb, d	5'- CTGGAGGAAATCCACGACATCGGATCCTGGCT G-3'
1157; PA4198 BamHI-up^d^	5'-CCAGAGGATCCAGCCGTTTTCGACGCAGT-3'
1158; PA4198 BamHI-down^d^	5'-CGAACACGTCGTTGAGCAGGATCCGCATG-3'
1218; fadDEc-HindIII-up^b^	5'-TCATAAGCTTGGGGTTGCGATGAC-3'
1251; fadD3-NdeI^b^	5'-AACCCATATGAATCCGTCCCCATCG-3'
1252; PA3568-Up-HindIII^d^	5'-ACTCCAAGCTTCACTCACTGCTTCATC-3'
1253; PA3568-Down-SalI^d^	5'-GGCTGGTCGACGAAGGCGTGTTGAA-3'
1254; PA1997-Up-BamHI^d^	5'-CCTGTGGATCCAGCAGATGCAGGA-3'
1255; PA1997-Down-SmaI^d^	5'-CTGAAGATGGCATTGTCG-3'
1256; PA0996-Up-BamHI^d^	5'-CTTCTTGCTTGGTTGCC-3'
1257; PA0996-Down-BamHI^d^	5'-CCAGCGGATCCTCCAGACACACATAGGA-3'
1258; PA2555-Up-HindIII^d^	5'-GCGTGAAGCTTCCGGCTACTCCATACA-3'
1259; PA2555-Down-KpnI^d^	5'-CCGCCGGTACCCAGGAACACTCGATTT-3'
1260; PA1617-Up-HindIII^d^	5'-CTAGGAAGCTTCTGGCGCAACGACTACAA-3'
1261; PA1617-Down-EcoRIb, c, d	5'-GTTCAGTTGCTCCAGGTC-3'
1441; PA1614-HindIII^c^	5'-GAAGCTTCATGACAGAGCAGCAAC-3'
1444; PA1617-NdeI^b^	5'-ATGCCATATGGTCACTGCAAATCGTCT-3'
2109; PA2893-upc, d	5'-GGCTATTTGCCGAAGTGC-3'
2110; PA3924-upc, d	5'-CGGATTCTATCTTGTGACC-3'

a Restriction enzyme sequences are underlined.

b Single copy complementation in *E. coli.*

c Single copy complementation in *P. aeruginosa.*

d *fadD* homologues cloning.

### Identification of *P. aeruginosa* Fatty acyl-CoA Synthetase Homologues

Potential *P. aeruginosa fadD* homologues were identified through BLAST [34] utilizing *E. coli* FadD sequence and alignment of *E. coli* FadD ATP/AMP [19], [35]–[37] and fatty acid binding motifs [38] with the FadD motifs of *P. aeruginosa fadD* homologues. Prediction of function of genes was obtained from Pseudomonas Genome Database (www.pseudomonas.com) [51]. PA2557, PA3860, and PA4198 were PCR amplified and cloned into pUC19 as BamHI fragments. The *fadD* homologues PA1617, PA1997, PA2555, PA3568, PA2893, and PA3924, were PCR amplified and cloned into pUC19 as HindIII/EcoRI, BamHI/SmaI, HindIII/KpnI, HindIII/SalI, and XbaI/BamHI fragments, respectively. For functional complementation testing, pUC19 vectors containing PAO1 *fadD* homologues were transformed into *E. coli fadD^−/^fadR^−^* strain (E2011) and the resulting transformants were patched onto 1x M9+1% (w/v) Brij-58+ ampicillin 100 µg/ml supplemented with 20 mM glucose, 0.2% (w/v) oleate (C_18∶1_^Δ9^), or decanoate (C_10∶0_).

### Single Copy Complementation of the E. coli *fadD^−/^fadR^−^* Mutant

To construct *fadD3*, *fadD5*, and *fadD6* single copy complementation vectors, first *fadD3*, *fadD5*, and *fadD6* PCR product were cloned into pET15b as NdeI/BamHI fragments. Next, the *fadD3-*His_6_, *fadD5-*His_6_, and *fadD6-*His_6_ BamHI/XbaI fragments were sub-cloned into miniTn7-Gm^r^ yielding miniTn7*-fadD3*, miniTn7-*fadD5* and miniTn7-*fadD6*. To construct the miniTn7-*fadD4,* first, the PCR product of *fadD4* was cloned into pET28a as NdeI/EcoRI fragment. The *fadD4-*His_6_ fragment, obtained by EcoRI digest, blunt-ending, and XbaI digest, was sub-cloned into miniTn7-Gm^r^ digested with the BamHI, blunt-ended and digested with XbaI. To construct miniTn7-*fadD_Ec_*, the *fadD_Ec_* PCR product was cloned as BamHI/blunt-end fragment into miniTn7-Gm^r^ digested with XbaI, blunt ended and digested BamHI.

Various miniTn7 vectors were integrated into E2011 using pTNS2 [52]. For the complementation study, two colonies of K-12, E2011, E2011/*att*Tn7::miniTn7-Gm^r^, E2011/*att*Tn7::*fadD_Ec_*, E2011/*att*Tn7::*fadD3*, E2011/*att*Tn7::*fadD4,* E2011/*att*Tn7::*fadD5,* and E2011/*att*Tn7::*fadD6* were patched onto 1x M9 medium +1% (w/v) Brij-58+0.25 mM isopropyl β-D-1-thiogalactopyranoside (IPTG) supplemented with 0.2% (w/v) FAs or 20 mM glucose. Plates were incubated for three days at 37°C and bacterial growth was scored from +1 to +6. Very little growth was marked as +1 and very heavy growth on a patch comparable to K12 on glucose at day three was marked as +6.

### Construction of Mutant Strains of PAO1

The *fadD3*, *fadD4*, *fadD5*, and *fadD6* gene replacement vectors were obtained as follows. pEX18T-*fadD3*-Gm^r^-*pheS_Pa_* was constructed by digesting pUC19-PA3860 with MscI and SgrAI, blunt-ending, and ligating it with Gm^r^-*pheS_Pa_*-*FRT* cassette that was SmaI excised from pwFRT-Gm^r^-*pheS_Pa_*. The PA3860-Gm^r^-*pheS_Pa_* fragment was excised from the resulting vector using BamHI and cloned into pEX18T. Similarly, pEX18T-*fadD4*-Gm^r^-*pheS_Pa_* was obtained by first sub-cloning *fadD4* gene as a HindIII/EcoRI fragment from pUC19-PA1617 into pEX18T, and *fadD4* was deactivated at the XhoI site by inserting the Gm^r^-*pheS_Pa_-mFRT* cassette SalI excised from pmFRT-Gm^r^-*pheS_Pa_*. pEX18T-*fadD5*-Gm^r^ was constructed by cloning *fadD5* PCR product (oligos #437 and #438) as BamHI/blunt-end fragment into pEX18T that was digested with BamHI and SmaI, and *fadD5* was deactivated at the blunt-ended XhoI site by inserting the Gm^r^-*FRT* cassette SmaI excised from pPS856. To construct pEX18T-*fadD6-*Gm^r^, *fadD6* PCR product (oligos #1093 and #512) was cloned as BamHI/blunt-end fragment into pEX18T that was digested with BamHI and SmaI, and *fadD6* was deactivated at the blunt-ended KpnI site by inserting the Gm^r^-*FRT* SmaI excised cassette from pPS856.

pEX18T-*fadD3*-Gm^r^-*pheS_Pa_*, pEX18T-*fadD4*-Gm^r^-*pheS_Pa_*, pEX18T-*fadD5*-Gm^r^, and pEX18T-*fadD6*-Gm^r^ gene replacement vectors were utilized as previously described [53] to obtain several mutant strains (P239, P243, P416, P677, P678, P685, P696, P698, P691, P722 P726, and P767). Unmarked mutations of *fadD* genes in various strains were obtained utilizing pFLP2 [53] or in one step via Flp mediated excision of Gm^r^-*pheS_Pa_*-*FRT* cassettes utilizing mutated version of *P. aeruginosa pheS* gene [54] and chlorinated phenylalanine (cPhe) counter-selection by transiently expressing *flp* on the non-replicative plasmid, pCD13SK-*flp-oriT*, as described previously [55]. Mutations transfer from strains P685, P239, P416 into PAO1, P678, P696, P698, and P722 were done as previously described [56], followed by Flp mediated excision of Gm^r^-*FRT* or Gm^r^-*pheS_Pa_*-*FRT* cassette, to obtain unmarked mutant strains P766, P768, P769, P770, P771, P772, P773, P969, and P972. Strain Δ*fadD3D4D5D6* (P781) was constructed in the PAO1-Δ*fadD3*::*FRT* background by subsequent transfer of mutation from strains P685, P239, and P416 followed by Flp mediated excision of Gm^r^-*FRT* or Gm^r^-*pheS_Pa_*-*FRT* cassette. Presence or absence of mutations of *fadD2D1*, *fadD3*, *fadD4*, *fadD5*, and *fadD6* in all mutant strains were confirmed by PCR (data not shown).

### Growth Phenotypes of Multiple *fadD* Mutants on Fatty Acids

To assess involvement of *P. aeruginosa fadD* homologues in FAs degradation, various strains (PAO1, double, triple, quadruple, quintuple, and sextuple *fadD* mutants) were purified on LB. After 24 h incubation at 37°C, two colonies of each strain were patched onto 1x M9 solid medium +1% (w/v) Brij-58 supplemented with 0.2% (w/v) FAs or 20 mM glucose. Plates were incubated at 37°C for four days. Growth of each strain was scored from +1 (little growth) to +6 (very heavy growth comparable to PAO1 on glucose at 96 h).

### Growth Curves Experiments

To further characterize various *fadD* mutants of *P. aeruginosa*, growth curve studies were performed using FAs as sole carbon source as described previously [14]. Doubling time of various strains in log-phase (Table S3) was calculated as follow: doubling time  = [0.301(t_2_-t_1_)]/(logOD_2_-logOD_1_) [57].

### Growth of *fadD* Mutants on Acyclic Terpenes

The Δ*fadD4*/*attB*::*fadD4* strain was constructed using a single copy complementation vector miniCTX2-*fadD4*, which was obtained by cloning the *fadD4* PCR product (oligos #1443 and #1261) as HindIII and EcoRI fragment into miniCTX2 and integrated into Δ*fadD4* mutant chromosome as described previously [58]. Stocks of citronellic (Sigma) and geranic acid (Sigma) (3% (w/v)) were prepared by neutralizing the compounds with equal molar sodium hydroxide and dissolving in 1% (w/v) Brij-58. PAO1 and various *fadD* mutants were grown overnight (14–16 h), starter culture were prepared as described by Kang *et al*. [14] and inoculated at 200-fold dilution into 1x M9 minimal medium +1% (w/v) Brij-58 supplemented with 0.1% (w/v) of citronellic acid, 0.1% (w/v) geranic acid or 20 mM glucose. Triplicate cultures were shaken at 30°C and optical densities were measured at day one and day six.

### Virulence Factors Production

Lipase, protease, phospholipase, and rhamnolipd productions by *fadD* mutants were tested as previously described [14].

### *In vitro* and *in vivo* Competition Studies

For *in vitro* and *in vivo* in competition studies, the Δ*fadD1D2D3D4D5D6* strain was complemented with *fadD2D1* and *fadD4* cloned into miniCTX2 and *fadD3*, *fadD5*, and *fadD6* cloned into miniTn7-Gm^r^. MiniCTX2-*fadD2D1D4* complementation vector, was constructed by cloning *fadD4* gene PCR product (oligos #1443 and #1261) as HindIII/blunt-end fragment into miniCTX2-*fadD2D1* digested with XhoI, blunt-ended and digested with HindIII. To construct miniTn7-*fadD3-fadD5-fadD6* vector, first *fadD3* was sub-cloned as BamHI fragment from pUC19-PA3890 into miniTn7-Gm^r^, resulting in miniTn7-PA3860. The *fadD6* was amplified with oligos #512 and #2210 and cloned as a BamHI/XbaI fragment into miniTn7-Gm^r^, resulting in miniTn7-PA3924. The *fadD5* was amplified with oligos #438 and #2109 and digested with BamHI, blunt-ended, and digested with XbaI. To construct the final vector, the miniTn7-PA3924 was digested with XbaI, blunt-ended and digested with NdeI and the 2.5 kb fragment (containing *fadD6*) was cloned simultaneously along with *fadD5* fragment into miniTn7-PA3860 digested with NdeI and SpeI. Integration of these plasmids into the *P. aeruginosa* chromosomes was performed as previously described ([58] and [52]).

The *in vitro* competition between Δ*fadD1D2D3D4D5D6* and its complement (strain P1021) on LB, or casamino acids (CAA), choline, glucose, glycerol, oleate (C_18∶1_^Δ9^) or PC was performed as described previously [14].

The *in vivo* competition study was performed as previously described [14]. Briefly, *mucA* was inactivated in the PAO1-Δ*fadD1D2D3D4D5D6* and its complement strains utilizing pUC18-'*mucA*'. Equal amounts of alginate overproducing sextuple mutant and its complement were resuspended in their own supernatants and mixed. Fourteen BALB/c mice were inoculated intratracheally with 6 x10^6^ colony forming units (CFU) of mixture of mutant (strain P973) and complement (strain P1028) as described previously [14]. At each time point (24 h and 48 h) seven mice were humanly euthanized, lungs were homogenized in 0.85% (w/v) saline and serial dilutions were plated on LB and LB+tetracycline 100 µg/ml to determine the total CFU and the complemented strain CFU. The competitive index (CI) was calculated as described [14].

## Supporting Information

Figure S1 **Alignment of motifs of potential fatty acyl-CoA synthetase homologues.** Amino acids with similar properties are assigned the same colors using CLC Sequence Viewer 6 software (www.clcbio.com).
(TIF)

Figure S2 **Growth phenotypes of various** ***fadD*** **homologues mutants on acyclic terpenes at day six.** Strains were grown in liquid 1x M9 medium +1% (w/v) Brij-58 supplemented with 0.1% (w/v) of citronellic acid or 0.1% (w/v) geranic acid at 30°C. Optical densities (ODs) of cultures were measured and compared to PAO1.
(TIF)

Table S1 **Potential FadD homologues of** ***P. aeruginosa*** **identified through BLAST and tested for complementation in** ***E. coli fadD^−/^fadR^−^*** **(E2011).**
(DOC)

Table S2 **Additional strains utilized in this study.**
(DOC)

Table S3 **Doubling time in minutes (min) of various strains in log-phase were calculated from growth curves in** **Fig. 2** **.**
(DOCX)
