# Supplementary figures and images for: Elucidating the Pseudomonas aeruginosa Fatty Acid Degradation Pathway: Identification of Additional Fatty Acyl-CoA Synthetase Homologues
Source: PLoS One. 2013 May 29;8(5):e64554. doi: 10.1371/journal.pone.0064554 (PMC3667196; doi:10.1371/journal.pone.0064554)

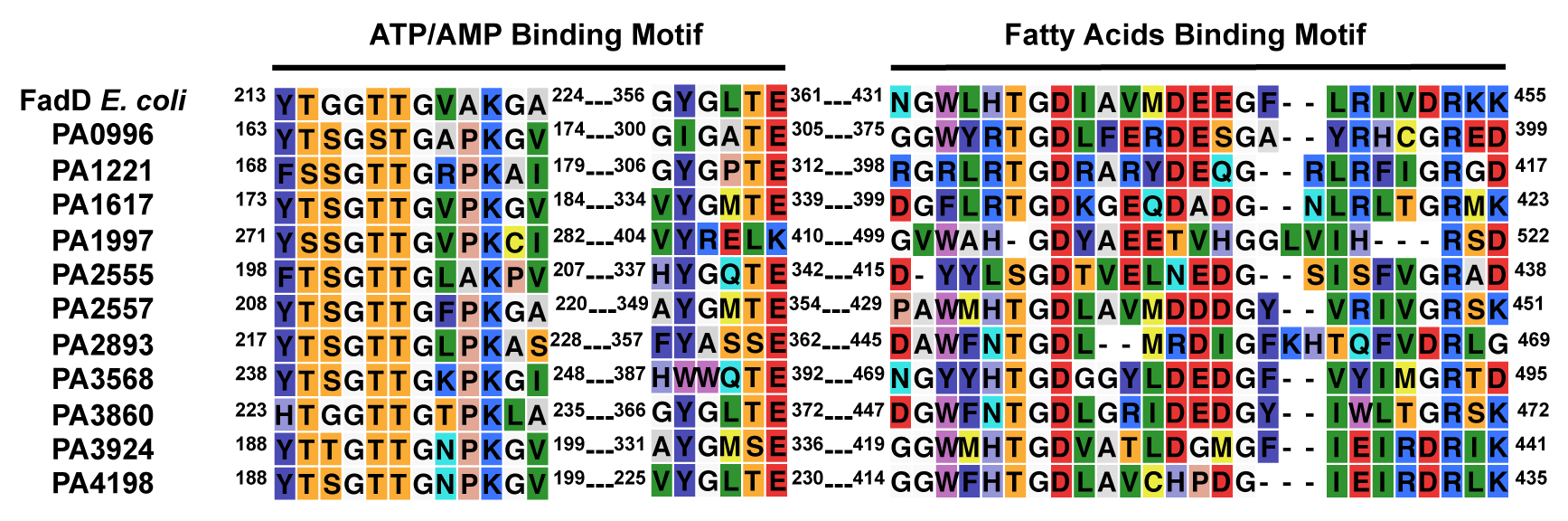

Supplement: Figure S1 — Alignment of motifs of potential fatty acyl-CoA synthetase homologues. Amino acids with similar properties are assigned the same colors using CLC Sequence Viewer 6 software (www.clcbio.com). (TIF) [file pone.0064554.s001.tif]

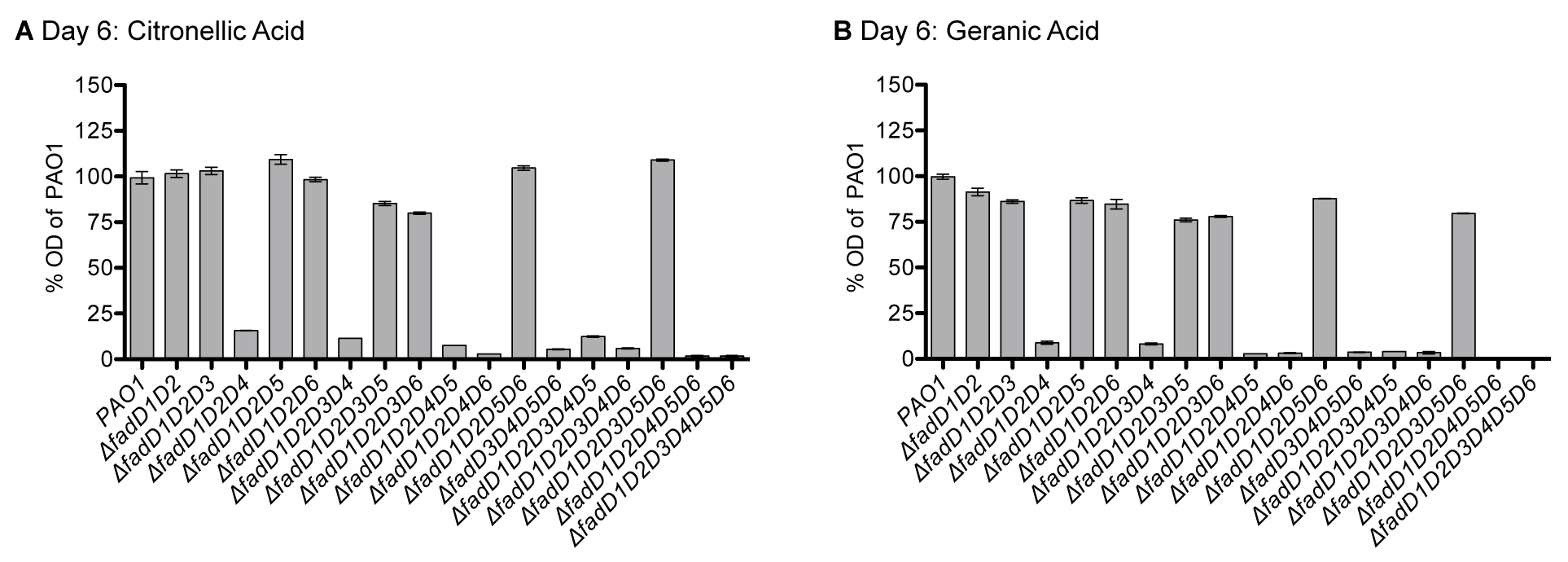

Supplement: Figure S2 — Growth phenotypes of various fadD homologues mutants on acyclic terpenes at day six. Strains were grown in liquid 1x M9 medium +1% (w/v) Brij-58 supplemented with 0.1% (w/v) of citronellic acid or 0.1% (w/v) geranic acid at 30°C. Optical densities (ODs) of cultures were measured and compared to PAO1. (TIF) [file pone.0064554.s002.tif]
