# Supplementary material for: Elucidating the Pseudomonas aeruginosa Fatty Acid Degradation Pathway: Identification of Additional Fatty Acyl-CoA Synthetase Homologues
Source: PLoS One. 2013 May 29;8(5):e64554. doi: 10.1371/journal.pone.0064554 (PMC3667196; doi:10.1371/journal.pone.0064554)
Supplement: Table S1 — Potential FadD homologues of P. aeruginosa identified through BLAST and tested for complementation in E. coli fadD−/fadR− (E2011). (DOC) [file pone.0064554.s003.doc]

**Table S1.** Potential FadD homologues of *P. aeruginosa* identified through BLAST and tested for complementation in *E. coli fadD^-^*/*fadR^-^* (E2011).

| **Gene** | **Predicted function^a^** | **Identity/ Similarity to *E. coli* FadD** | **Length (amino acids residues)^b^** | **Growth of E2011 on oleate^c^** | **Growth of E2011 on decanoate^c^** |
| --- | --- | --- | --- | --- | --- |
| PA0996 | probable coenzyme A ligase | 28%/43% | 517 | - | - |
| PA1617 | probable AMP-binding enzyme | 24%/40% | 555 | +4 | +1 |
| PA1221 | hypothetical protein | 24%/40% | 618 | - | - |
| PA1997 | probable AMP-binding enzyme | 21%/37% | 651 | - | - |
| PA2555 | probable AMP-binding enzyme | 25%/44% | 555 | - | - |
| PA2557 | probable AMP-binding enzyme | 31%/52% | 564 | - | - |
| PA2893 | very long-chain acyl-CoA synthetase | 22%/37% | 608 | +4 | +1 |
| PA3568 | probable acetyl-CoA synthetase | 24%/39% | 628 | - | - |
| PA3860 | probable AMP-binding enzyme | 29%/47% | 632 | +4 | +1 |
| PA3924 | probable medium-chain acyl-CoA ligase | 24%/43% | 560 | +4 | +1 |
| PA4198 | probable AMP-binding enzyme | 26%/43% | 540 | - | - |

^a^ From [www.pseudomonas.com](http://www.pseudomonas.com/)

^b^ *E. coli* FadD is 561 amino acids (aa) long, FadD1 (PA3299, not shown) is 562 aa, and FadD2 (PA3300, not shown) is 562 aa. ^c^Genes were cloned into pUC19 and tested in E2011 (*E. coli fadD^-^*/*fadR^-^*) on 1x M9 medium + 1% (w/v) Brij-58 + ampicillin 100 μg/ml + 0.2% (w/v) oleate (C_18:1_^∆9^) or decanoate (C_10:0_) as sole carbon source  +1 is very little growth whereas +6 is very heavy growth comparable to K12 on glucose at day 3.

.
