# Supplementary material for: Elucidating the Pseudomonas aeruginosa Fatty Acid Degradation Pathway: Identification of Additional Fatty Acyl-CoA Synthetase Homologues
Source: PLoS One. 2013 May 29;8(5):e64554. doi: 10.1371/journal.pone.0064554 (PMC3667196; doi:10.1371/journal.pone.0064554)
Supplement: Table S2 — Additional strains utilized in this study. (DOC) [file pone.0064554.s004.doc]

**Table S2.** Additional strains utilized in this study.

| Strain | **Lab ID** | **Relevant Properties** | **Source/ reference** |
| --- | --- | --- | --- |
| ***E. coli*** |  |  |  |
| EPMax10B-*lacIq*/*pir* | E1869 | F- λ*- mcrA* Δ(*mrr-hsdRMS-mcrBC*) ϕ80d*lacZ* Δ*M15* Δ*lacX74 deoR recA1 endA1* *araD139* Δ*(ara, leu) galU galK rpsL nupG lacIq-FRT8 pir-FRT4* | Available lab strain |
| EPMax10B-Δ*dapA*::*pir-lacIq-*Gmr/*mob*-Kmr/*leu*+ | E2072 | Gmr, Kmr;F- λ*- mcrA* Δ(*mrr-hsdRMS-mcrBC*) ϕ80d*lacZ*Δ*M15* Δ*lacX74 deoR recA1 endA1galU galK rpsL nupG* Δ*dapA*::*pir-lacIq*-Gmr-*FRT8 mob*[*recA*::*RP4-2 Tc*::*Mu*-Kmr] *leu*+ | Available lab strain |
| EPMax10B-*pir116*/Δ*asd*/*mob*-Kmr | E1353 | Kmr;F*-* λ*- mcrA* Δ(*mrr-hsdRMS-mcrBC*) ϕ80d*lacZ* Δ*M15* Δ*lacX74 deoR recA1 endA1 araD139* Δ*(ara, leu)7697 galU galK rpsL nupG Tn-pir116-FRT2* Δ*asd*::*FRT mob*[*recA*::*RP4-2 Tc*::*Mu*-Kmr] | Available lab strain |
| HPS1-*mob*-Kmr/Δ*asd*/ *pir116* | E0463 | Cmr, Kmr, Tcr;*e14*- (*mcrA*) *recA1 endA1* *gyrA96* *thi*-1 *hsdR17* *supE44* *relA1* Δ(*lac-proAB*) *rif zxx*::miniTn*5Lac4* (*lacIq lacZ*Δ*M15*) Δ*asd::FRT uidA*::*pir116 mob*[*recA*::*RP4-2 Tc*::*Mu*-Kmr] | ­Available lab strain |
| ***P. aeruginosa*** |  |  |  |
| Δ*fadD3* | P677 | PAO1-Δ*fadD3*::*FRT* | This study |
| *fadD4*::Gmr | P685 | Gmr; PAO1-*fadD4*::Gmr-*pheSPa*-*mFRT* | This study |
| Δ*fadD5* | P243 | PAO1-*fadD5*::*FRT* | This study |
| Δ*fadD6* | P972 | PAO1-*fadD6*::*FRT* | This study |
| Δ*fadD1D2*/*fadD5*::Gmr | P239 | Gmr; PAO1-Δ*fadD2D1*::*FRT*/*fadD5*::Gmr-*FRT* | This study |
| Δ*fadD1D2D5*/ *fadD6*::Gmr | P416 | Gmr; PAO1-Δ*fadD2D1*::*FRT*/*fadD5*::*FRT*/ *fadD6*::Gmr-*FRT* | This study |

Abbreviations:

Cmr, chloramphenicol resistance.
