# Supplementary material for: Elucidating the Pseudomonas aeruginosa Fatty Acid Degradation Pathway: Identification of Additional Fatty Acyl-CoA Synthetase Homologues
Source: PLoS One. 2013 May 29;8(5):e64554. doi: 10.1371/journal.pone.0064554 (PMC3667196; doi:10.1371/journal.pone.0064554)
Supplement: Table S3 — Doubling time in minutes (min) of various strains in log-phase were calculated from growth curves in Fig. 2 . (DOCX) [file pone.0064554.s005.docx]

**Table S3.** Doubling time in minutes (min) of various strains in log-phase were calculated from growth curves in Fig. 2.

|  | **Doubling time (min)** | | | | |
| --- | --- | --- | --- | --- | --- |
| **Strain** | C_6:0_ | C_10:0_ | C_14:0_ | C_18:1_^Δ9^ | Glucose |
| PAO1 | 81 | 90 | 106 | 107 | 57 |
| *∆fadD1D2* | 240 | 213 | 225 | 374 | 56 |
| *∆fadD1D2D4* | 0 | 386 | 241 | 1131 | 57 |
| *∆fadD3D4D5D6* | 81 | 122 | 132 | 101 | 57 |
| *∆fadD1D2D3D5D6* | 288 | 327 | 206 | 397 | 56 |
| *∆fadD1D2D3D4D5D6* | 0 | 0 | 0 | 0 | 55 |

doubling time=[0.301(t_2_-t_1_)]/(logOD_2_-logOD_1_)
